# Severity‐Graded MRI‐Detected TMJ Effusion Is Associated With Pain and Dysfunction in Temporomandibular Disorder Patients

**DOI:** 10.1111/joor.70276

**Published:** 2026-07-25

**Authors:** Yeon‐Hee Lee, Seong‐Woo Jang, Seonggwang Jeon, Q‐Schick Auh, Yung‐Kyun Noh

**Affiliations:** ^1^ School of Dentistry, Dental Research Institute Seoul National University Seoul Republic of Korea; ^2^ Department of Orofacial Pain and Oral Medicine Kyung Hee University Dental Hospital Seoul Republic of Korea; ^3^ Equipment Technology Group Samsung SDI Cheongju–si, Chungcheongbuk–do Republic of Korea; ^4^ Department of Computer Science Hanyang University Seoul Republic of Korea; ^5^ Affiliate Professor, School of Computational Sciences Korea Institute for Advanced Study (KIAS) Seoul Republic of Korea

**Keywords:** arthralgia, effusion, machine learning, magnetic resonance imaging, pain, temporomandibular disorder, temporomandibular joint

## Abstract

**Background:**

Temporomandibular joint (TMJ) effusion on magnetic resonance imaging (MRI) is considered a radiologic sign of intra‐articular inflammation; however, its clinical relevance to pain and mandibular dysfunction remains inconsistent.

**Objective(s):**

The objective of this study is to determine whether severity‐graded MRI‐detected TMJ effusion is associated with pain intensity and dysfunction in patients with temporomandibular disorders (TMD).

**Methods:**

This retrospective clinical imaging study included 221 patients with TMJ arthralgia diagnosed according to the Diagnostic Criteria for TMD (DC/TMD Axis I). TMJ effusion was assessed using 3.0‐T MRI and classified as Grade 0 (non‐effusion), Grade 1 (mild effusion), or Grade 2 (moderate‐to‐severe effusion). Pain and dysfunction were evaluated using the visual analog scale (VAS), dysfunction index (DI), palpation index (PI), craniomandibular index (CMI), symptom duration, and associated clinical symptoms. Group comparisons and multivariable logistic regression analyses were performed.

**Results:**

Compared with the non‐effusion group, the effusion group showed significantly higher VAS (5.08 ± 2.16 vs. 3.45 ± 2.09, *p* < 0.001), DI (0.29 ± 0.24 vs. 0.21 ± 0.23, *p* = 0.014), and CMI (0.27 ± 0.26 vs. 0.18 ± 0.24, *p* = 0.025), as well as longer symptom duration (17.25 ± 32.34 vs. 6.34 ± 11.89 months, *p* < 0.001). Across effusion grades, VAS, DI, PI, and CMI increased significantly in a dose–response manner (all *p* < 0.01). In multivariable analyses, female sex (OR, 2.78; 95% CI, 1.55–4.97), higher VAS (OR, 2.03; 95% CI, 1.51–2.72), higher CMI (OR, 1.68; 95% CI, 1.26–2.25), TMJ noise (OR, 2.05; 95% CI, 1.17–3.59), locking (OR, 2.45; 95% CI, 1.39–4.30), and tinnitus (OR, 2.28; 95% CI, 1.30–4.02) were independently associated with effusion presence. Female sex (OR, 3.29; 95% CI, 1.47–7.38), higher VAS (OR, 2.11; 95% CI, 1.46–3.04), and higher CMI (OR, 1.71; 95% CI, 1.24–2.36) were independently associated with Grade 2 effusion.

**Conclusions:**

Severity‐graded MRI‐detected TMJ effusion is associated with greater pain intensity and dysfunction in patients with TMJ arthralgia, supporting its interpretation as a clinically meaningful imaging marker rather than an incidental radiologic finding.

## Graphical abstract

In patients with TMJ arthralgia, MRI‐detected joint effusion was graded from Grade 0 (no effusion) to Grade 2 (moderate‐to‐severe effusion) using T2‐weighted and proton density‐weighted images. Increasing effusion severity was associated with greater pain intensity and mandibular dysfunction. These associations remained evident after considering concomitant anterior disc displacement and osteoarthritic changes, supporting TMJ effusion severity as a clinically meaningful imaging marker rather than an incidental fluid finding.

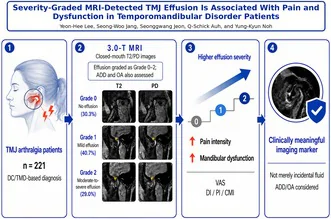

## 1 Introduction

Temporomandibular disorders (TMD) encompass diverse conditions affecting the temporomandibular joint (TMJ), masticatory muscles, and associated structures, often resulting in pain, joint noise, and limited mandibular function [1, 2]. TMD is a common musculoskeletal pain disorder involving the orofacial region, affecting approximately 31% of adults, with women being 1.5 to 2.24 times more likely to be affected than men [3, 4]. However, the clinical significance of TMJ inflammation, particularly TMJ effusion, in relation to TMD pain remains unclear, posing challenges for both clinicians and researchers.

Among imaging findings, TMJ effusion refers to intra‐articular fluid signal within the TMJ space—typically visualized as high signal intensity on fluid‐sensitive magnetic resonance imaging (MRI) sequences—reflecting an imbalance between synovial fluid production and clearance [5]. Conceptually, TMJ effusion is thought to arise from intra‐articular inflammation (e.g., synovitis), increased vascular permeability, and biochemical changes within the joint environment [6]. The fluid may represent synovial fluid with inflammatory exudative components and can be accompanied by elevated inflammatory mediators and tissue breakdown products [7], plausibly linking effusion to nociceptive sensitization and pain.

In larger synovial joints—particularly the knee and hip—the link between imaging‐defined effusion/effusion‐synovitis and pain has been studied extensively. In general, greater effusion‐synovitis burden tends to accompany symptomatic disease [8]. For the knee, effusion‐related findings are often regarded as markers of active joint pathology, although these abnormalities may relate more consistently to structural osteoarthritis (OA) severity than to pain itself [9, 10]. However, the results are not fully consistent [11]: associations with pain severity are often modest and can weaken after accounting for coexisting structural lesions and clinical factors, and some evidence suggests that synovial inflammation (synovitis) tracks pain fluctuations more reliably than “fluid alone.” For the hip, the evidence base is smaller and more heterogeneous, with mixed findings across cohorts and protocols [12, 13]. A major reason for disagreement is measurement variability, especially on non‐contrast MRI, where joint fluid and synovial thickening are difficult to separate and are frequently combined as “effusion‐synovitis,” complicating mechanistic interpretation and contributing to inconsistent conclusions.

In contrast, evidence regarding TMJ effusion and TMD pain intensity in TMJ arthralgia patients remains comparatively limited and, where available, inconsistent. Reported variability may reflect differences in diagnostic frameworks, MRI acquisition protocols, effusion definitions and grading criteria, timing of imaging relative to symptom fluctuations, and the presence of coexisting intra‐articular conditions (e.g., disc displacement, osteoarthritic changes) and patient‐level factors (e.g., sex, symptom duration, and related clinical features) [14, 15, 16]. As a result, the clinical meaning of TMJ effusion—particularly whether it reliably tracks with pain severity and whether greater effusion corresponds to greater pain—remains insufficiently clarified.

Therefore, this study aimed to evaluate the clinical relevance of severity‐graded MRI‐detected TMJ effusion in patients with TMD. Specifically, we examined whether pain intensity and dysfunction‐related indices differ according to the presence of TMJ effusion and whether increasing effusion severity demonstrates a dose–response relationship with pain and functional burden. We hypothesized that both the presence and greater severity of TMJ effusion would be associated with higher pain intensity and greater functional impairment. By integrating standardized MRI assessment, Diagnostic Criteria for Temporomandibular Disorders (DC/TMD)‐based clinical diagnosis, pain and dysfunction indices, and exploratory classification analyses, this study sought to clarify whether TMJ effusion should be interpreted as a clinically meaningful imaging marker rather than a simple incidental radiologic finding.

## 2 Methods

### 2.1 Ethics Statement

This study was conducted in accordance with the principles of the Declaration of Helsinki and was approved by the Institutional Review Board of Kyung Hee University Dental Hospital, Seoul, South Korea (KHD IRB, No. KH‐DT20030‐1). Written informed consent was obtained from all participants before inclusion in the study.

### 2.2 Study Population

A total of 221 patients with TMJ arthralgia (148 women and 73 men; mean age, 36.03 ± 17.96 years) who visited Kyung Hee University Dental Hospital between January 2022 and February 2025 were enrolled. TMJ arthralgia was diagnosed according to the DC/TMD, Axis I [17] and was defined as pain of joint origin modified by jaw movement or function and reproduced by TMJ provocation tests, including palpation or jaw movements.

Based on MRI findings, patients were classified into a non‐effusion group and an effusion group. Patients with unilateral or bilateral TMJ effusion were assigned to the effusion group, whereas those without effusion in either joint were assigned to the non‐effusion group. Of the 221 patients, 67 (30.3%) were classified into the non‐effusion group and 154 (69.7%) into the effusion group. For severity‐based analyses, effusion was further categorized into Grade 0 (non‐effusion), Grade 1 (mild effusion), and Grade 2 (moderate‐to‐severe effusion). Among the 154 patients with effusion, 90 (58.4%) were classified as Grade 1 and 64 (41.6%) as Grade 2.

### 2.3 MR Image Acquisition

High‐resolution MRI was performed using a 3.0‐T MRI system (Signa Genesis, GE Healthcare, Chicago, IL, USA) with a 6‐cm × 8‐cm surface coil according to the institutional TMJ imaging protocol. All scans included sagittal oblique sections with a section thickness of ≤ 3 mm, field of view of 15 cm, and matrix size of 256 × 224. Spin‐echo sagittal MRI images were obtained based on axial localizer images. Proton density–weighted and T2‐weighted images were acquired using repetition time/echo time parameters of 650/14 and 2650/82, respectively, as described previously [2, 18]. T1‐weighted, T2‐weighted, and proton density–weighted images were obtained in a natural closed‐mouth position. In addition, proton density–weighted dynamic views acquired sequentially during mouth opening were available and were used as supportive information for assessing anterior disc displacement.

### 2.4 MRI Assessment of TMJ Effusion

On MRI, TMJ effusion was defined as an abnormal intra‐articular fluid collection visualized as a hyperintense signal on T2‐weighted images and corroborated on proton density–weighted sequences. Effusion grading was performed using representative closed‐mouth T2‐weighted and proton density–weighted images, because intra‐articular fluid is most consistently visualized on these fluid‐sensitive sequences. Dynamic proton density–weighted opening views were not used for effusion grading but were referenced when evaluating anterior disc displacement. Effusion was assessed on a joint‐by‐joint basis by identifying a definite fluid signal within the superior and/or inferior joint compartment that exceeded minimal physiologic fluid and formed a discernible fluid layer or collection. For patient‐level grouping, unilateral or bilateral effusion was classified as effusion presence, whereas absence of effusion in both joints was classified as non‐effusion. Effusion severity was graded using an ordinal scale adapted from previous MRI‐based TMJ effusion grading systems [14, 19, 20]. Grade 0 indicated no definite fluid signal beyond minimal physiologic fluid; Grade 1 indicated a small but definite linear or focal fluid signal without clear joint distension; and Grade 2 indicated a more prominent fluid collection occupying a larger portion of the joint space, often accompanied by apparent intra‐articular distension. In patients with bilateral effusion, the higher grade was used for patient‐level severity classification. In addition to effusion, anterior disc displacement (ADD) and OA were assessed on the same MRI examination and recorded as present or absent [21]. MRI‐based effusion grading and assessment of ADD/OA were independently performed by two experienced orofacial pain specialists (Y.‐H.L. and Q.‐S.A.). Inter‐rater reliability was excellent, with Cohen's kappa values ranging from 0.83 to 0.89. Any discrepancies were resolved through repeated joint review and consensus discussion.

### 2.5 Clinical Variables

The following clinical variables were collected from all patients. Pain intensity was assessed using a **visual analog scale (VAS)** ranging from 0 to 10, where 0 indicated no pain and 10 indicated the worst imaginable pain [22]. TMJ dysfunction was evaluated using the **Craniomandibular Index (CMI)**, which is derived from the average of the **Dysfunction Index (DI)** and **Palpation Index (PI)**. DI reflects functional impairment of the TMJ, whereas PI reflects tenderness of the masticatory muscles and TMJ on palpation [23]. **Symptom duration** was defined as the interval between the patient's first recognition of TMJ pain and the initial clinic visit, expressed in months [23]. The following patient‐reported or clinically documented symptoms were recorded dichotomously (yes/no): **TMJ noise**, **locking**, **muscle stiffness**, **tinnitus**, and **bruxism**. TMJ noise included clicking, popping, or crepitus during mandibular movement. Locking was defined as a patient‐reported inability to open or close the mouth normally. Muscle stiffness referred to stiffness, heaviness, or discomfort in the jaw or facial muscles at rest or during function. Tinnitus was defined as the perception of ringing or buzzing without an external source. Bruxism referred to awake or sleep‐related clenching or grinding of the teeth [24].

### 2.6 Sample Size

The required sample size was estimated using G*Power version 3.1.9.7 (Heinrich‐Heine‐Universität Düsseldorf, Düsseldorf, Germany). A minimum of 134 participants was required to achieve a statistical power of 0.95 at a significance level of 0.05 for detecting group differences in the primary outcome. The final sample of 221 patients exceeded this minimum requirement.

### 2.7 Statistical Analysis

All statistical analyses were performed using **IBM SPSS Statistics for Windows, version 26.0** (IBM Corp., Armonk, NY, USA), along with **Python‐based regression and classification analyses**. TMJ effusion was analysed in three ways: (1) **binary effusion presence** (non‐effusion vs. effusion), (2) **three‐level effusion severity** [Grade 0 (non‐effusion), Grade 1 (mild effusion), and Grade 2 (moderate‐to‐severe effusion)], and (3) **severity‐focused binary classification** [Grade 2 vs. Grades 0–1].

Continuous variables are presented as mean ± standard deviation, and categorical variables are presented as frequency (percentage). For comparisons between the non‐effusion and effusion groups, **Student's *t*‐test** was used for continuous variables and the **χ** ^**2**^ **test** (or **Fisher's exact test**, when appropriate) for categorical variables. For comparisons across the three severity groups, **one‐way analysis of variance (ANOVA)** was used for continuous variables and the **χ** ^**2**^ **test** for categorical variables. When appropriate, multiplicity‐adjusted *p* values were calculated using **Bonferroni correction**. To quantify the strength of associations between categorical variables and effusion status, **Cramer's V** was calculated. All tests were two‐sided, and *p* < 0.05 was considered statistically significant.

To identify factors associated with TMJ effusion presence, univariable and multivariable binary logistic regression analyses were performed using Python‐based regression models (Python version 3.12.13; scikit‐learn version 1.6.1; statsmodels version 0.14.6; imbalanced‐learn version 0.14.1; and SciPy version 1.16.3), and the results are presented as odds ratios (ORs), 95% confidence intervals (CIs), and *p* values. Additional univariable and multivariable binary logistic regression analyses were performed to identify factors associated with **moderate‐to‐severe effusion (Grade 2)**, using **Grades 0–1 as the reference group**. These analyses were used to evaluate clinical variables associated with both effusion presence and higher effusion severity. To assess whether concomitant structural MRI findings influenced the effusion–pain/dysfunction relationship, a sensitivity analysis was performed in which ADD and OA were added as covariates to linear regression models relating effusion, modelled as both presence and grade, to pain and dysfunction measures (VAS and CMI), with adjustment for age and sex. Multicollinearity was evaluated using variance inflation factors (VIFs). Unilateral effusion, defined as right‐ or left‐sided effusion, and bilateral effusion were compared with respect to VAS, DI, PI, and CMI using the Mann–Whitney U test. A three‐group comparison among no effusion, unilateral effusion, and bilateral effusion was performed using the Kruskal–Wallis test.

### 2.8 Exploratory Machine Learning and Classification Analyses

Because clinical symptoms and dysfunction indices in TMD are often interrelated, the exploratory modelling analyses were performed to complement the primary clinical association analyses. Specifically, these analyses were used to identify clinical features independently or jointly associated with MRI‐detected effusion, to examine their relative contribution, and to quantify how well a routinely collected clinical feature set could discriminate effusion using the area under the receiver operating characteristic curve (AUROC). These analyses were treated as exploratory and hypothesis‐generating and were not intended to replace the primary clinical analyses or to establish a deployable prediction model.

Exploratory Python‐based machine learning analyses were conducted to assess the discriminative performance of clinical variables for predicting MRI‐detected TMJ effusion. Two primary prediction tasks were defined: (1) binary classification of TMJ effusion presence (non‐effusion vs. effusion) and (2) multiclass classification of TMJ effusion severity [Grade 0, Grade 1, and Grade 2].

For both tasks, the dataset was divided into 80% training and 20% testing subsets using stratified sampling to preserve class proportions. Missing values were imputed as 0 according to the predefined analytic pipeline. All input features were standardized using StandardScaler, with parameters derived from the training set and applied to the test set.

For binary classification of effusion presence, a logistic regression model was trained with a maximum iteration setting of 1000. For multiclass classification of effusion severity, a one‐vs‐rest multiclass logistic regression model was used to estimate class‐specific probabilities for Grades 0, 1, and 2. Model performance was evaluated using receiver operating characteristic (ROC) curve analysis and AUROC. For binary classification, AUROC and its 95% CI were calculated. For multiclass classification, class‐specific, macro‐averaged, and micro‐averaged AUROCs were obtained. Confidence intervals for AUROC were estimated using bootstrap resampling with 2000 iterations.

To explore the impact of class imbalance, the synthetic minority over‐sampling technique (SMOTE) was applied only to the training data. In the binary setting, the non‐effusion group was oversampled to match the effusion group. In the three‐level setting, minority classes were oversampled to match the size of the largest class. In addition, four binary classification tasks derived from ordinal effusion grades were evaluated: Grade 0 vs. Grades 1–2, Grades 0–1 vs. Grade 2, Grade 0 vs. Grade 1, and Grade 0 vs. Grade 2. For each task, baseline and SMOTE‐applied models were compared. Differences in AUROC between models were assessed using the DeLong test. These analyses were performed as exploratory and sensitivity analyses to evaluate the robustness of predictive performance under different class‐balance conditions.

## 3 Results

### 3.1 Study Population and Distribution of MRI‐Detected TMJ Effusion

A total of 221 patients with TMJ arthralgia were included, with a female‐to‐male ratio of 2.03:1 (148 women and 73 men). Of these, 67 patients (30.3%) were classified into the non‐effusion group and 154 patients (69.7%) into the effusion group. When stratified by effusion severity in the overall cohort, 67 patients (30.3%) were classified as Grade 0 (non‐effusion), 90 (40.7%) as Grade 1 (mild effusion), and 64 (29.0%) as Grade 2 (moderate‐to‐severe effusion). Within the effusion group, Grade 1 and Grade 2 accounted for 58.4% and 41.6%, respectively. Representative MRI findings for each effusion grade on T2‐weighted and proton density‐weighted sequences are shown in Figure 1. Mean age did not differ significantly between the non‐effusion and effusion groups (35.70 ± 18.42 vs. 36.17 ± 17.82 years, *p* = 0.861), and sex distribution also did not differ significantly in the binary comparison (*p* = 0.087). Across the three severity groups, age remained similar (*p* = 0.997), whereas sex distribution showed a significant trend, with greater female predominance at higher effusion grades (*p* = 0.026) (Tables 1 and 2).

**FIGURE 1:**
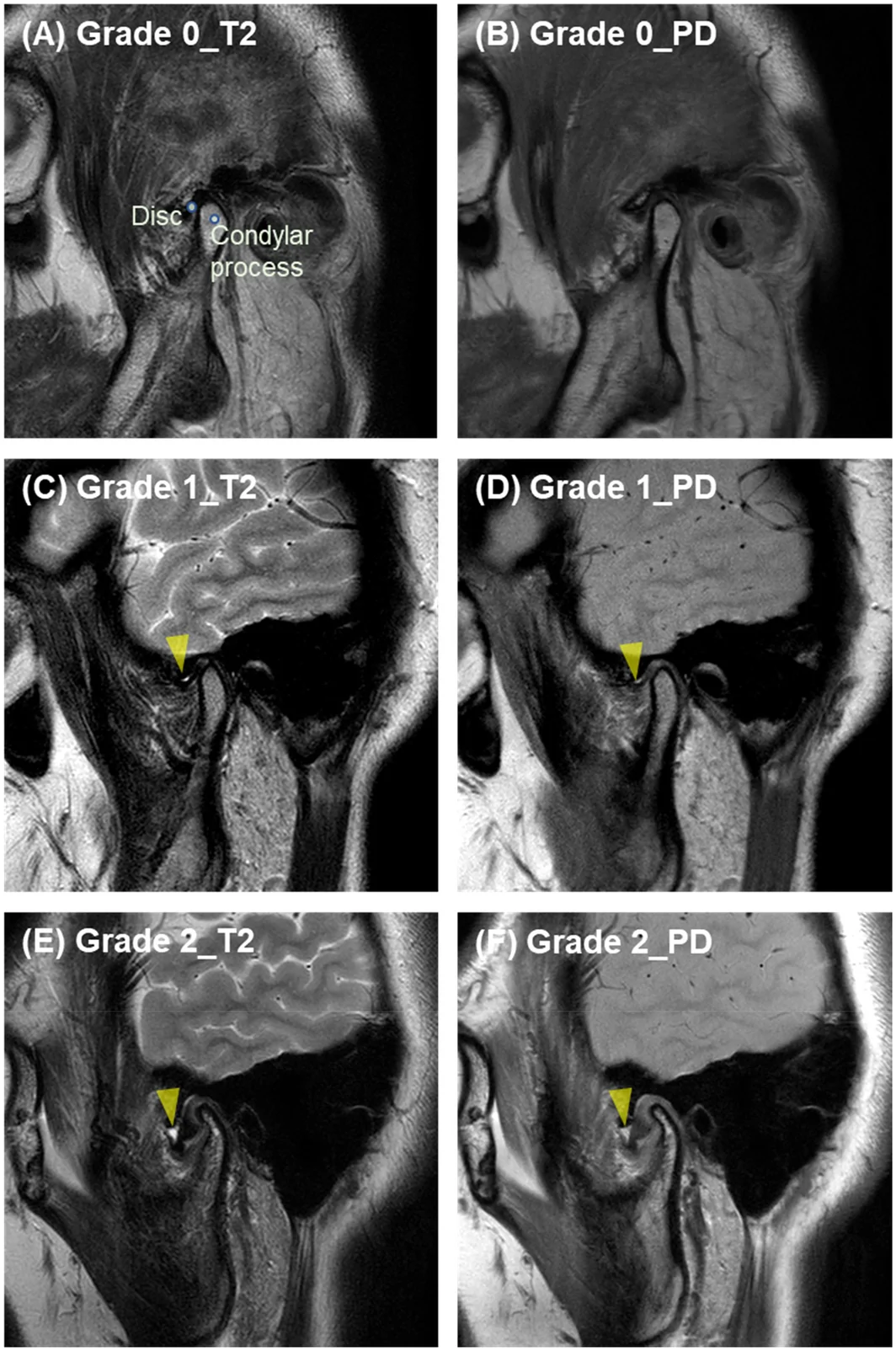
Representative MRI images of TMJ effusion grades on T2‐weighted and proton density–weighted sequences. Representative sagittal oblique magnetic resonance images illustrating the three grades of temporomandibular joint (TMJ) effusion. (A, B) Grade 0 (non‐effusion), showing no definite intra‐articular fluid signal. (C, D) Grade 1 (mild effusion), showing a small but definite fluid signal within the joint space (arrowheads). (E, F) Grade 2 (moderate‐to‐severe effusion), showing a more prominent intra‐articular fluid collection occupying a larger portion of the joint space (arrowheads). (A, C, E) T2‐weighted images; (B, D, F) proton density–weighted images. In panel A, the disc and condylar process are indicated for anatomical reference.

**TABLE 1:** Demographic, pain‐related, and clinical characteristics of patients according to tMJ effusion status.

Parameters	Non‐effusion (*n* = 67)	Effusion (*n* = 154)	*p*	adjusted *p*
	mean ± SD or *n* (%)	mean ± SD or *n* (%)		
*Demographics*				
Age (years)^a^	35.70 ± 18.42	36.17 ± 17.82	0.861	0.861
Sex^b^				
[male]	28 (41.8%)	45 (29.2%)	0.087	0.091
[female]	39 (58.2%)	109 (70.8%)		
***Pain characteristics***				
VAS^a^	3.45 ± 2.09	5.08 ± 2.16	**< 0.001** ***	**< 0.001** ***
DI^a^	0.21 ± 0.23	0.29 ± 0.24	**0.014** *	**0.02155** *
PI^a^	0.16 ± 0.27	0.24 ± 0.30	**0.048** *	0.054
CMI^a^	0.18 ± 0.24	0.27 ± 0.26	**0.025** *	**0.032** *
Symptom duration (month)^a^	6.34 ± 11.89	17.25 ± 32.34	**< 0.001** ***	**< 0.001** ***
***MRI findings***				
Effusion severity				
[absence]	67 (100%)	0 (0.0%)	**< 0.001** ***	**< 0.001** ***
[mild]	0 (0.0%)	90 (58.4%)		
[moderate to severe]	0 (0.0%)	64 (41.6%)		
***Clinical characteristics***				
TMJ noise^b^				
[absence]	34 (50.7%)	49 (31.8%)	**0.010** *	**0.021** *
[presence]	33 (49.3%)	105 (68.2%)		
Locking^b^				
[absence]	48 (71.6%)	76 (49.4%)	**0.003** **	**0.016** *
[presence]	19 (28.4%)	78 (50.6%)		
Muscle stiffness^b^				
[absence]	41 (61.2%)	75 (48.7%)	0.107	0.1181
[presence]	26 (38.8%)	79 (51.3%)		
Tinnitus^b^				
[absence]	48 (71.6%)	106 (68.8%)	0.654	0.799
[presence]	19 (28.4%)	48 (31.2%)		
Bruxism^b^				
[absence]	53 (79.1%)	116 (75.3%)	0.746	0.821
[presence]	14 (20.9%)	38 (24.7%)		

*Note:* Data are presented as mean ± SD or *n* (%). Bonferroni‐adjusted *p* values are presented in the final column. Statistical significance was defined as *p* < 0.05. Bold indicates statistical significance.

Abbreviations: CMI, craniomandibular index; DI, dysfunction index; PI, palpation index; SD, standard deviation; TMD, temporomandibular disorder; TMJ, temporomandibular joint; VAS, visual analog scale.

^a^ Continuous variables were analysed using the Student's *t*‐test.

^b^ Categorical variables were analysed using the χ^2^ test.

* *p* < 0.05.

** *p* < 0.01.

*** *p* < 0.001.

**TABLE 2:** Clinical characteristics by tMJ effusion grade and multiclass classification coefficients.

Variable	Group comparison				Regression coefficient		
	Grade 0 (non‐effusion) (*n* = 67)	Grade 1 (mild effusion) (*n* = 90)	Grade 2 (moderate‐to‐severe effusion) (*n* = 64)	*p*	Grade 0 coefficient	Grade 1 coefficient	Grade 2 coefficient
Age^a^	35.70 ± 18.42	36.02 ± 17.68	36.38 ± 18.15	0.997	0.150902	0.077031	−0.22334
Sex (Female)^b^	39 (58.2%)	58 (64.4%)	51 (79.7%)	**0.026** *	−0.321188	0.020225	0.340321
VAS^a^	3.45 ± 2.09	4.72 ± 2.22	5.59 ± 1.99	**< 0.001** ***	−0.876544	−0.030141	0.88061
DI^a^	0.21 ± 0.23	0.25 ± 0.20	0.35 ± 0.28	**0.003** **	−0.605885	0.091674	0.326785
PI^a^	0.16 ± 0.27	0.19 ± 0.23	0.31 ± 0.37	**0.005** **	0.224831	−0.160146	0.059775
CMI^a^	0.18 ± 0.24	0.22 ± 0.21	0.33 ± 0.32	**0.003** **	−0.150101	−0.049677	0.184862
Symptom duration^a^	6.34 ± 11.90	21.71 ± 38.18	10.96 ± 20.27	**0.002** **	−0.684784	0.563037	−0.360786
TMJ noise^b^	33 (49.3%)	61 (67.8%)	44 (68.8%)	**0.028** *	−0.258423	0.041624	0.169718
Locking^b^	19 (28.4%)	44 (48.9%)	34 (53.1%)	**0.008** **	−0.414157	0.067316	0.231449
Muscle stiffness^b^	26 (38.8%)	43 (47.8%)	36 (56.2%)	0.135	−0.263979	−0.114052	0.366493
Tinnitus^b^	11 (16.4%)	36 (40.0%)	24 (37.5%)	**0.004** **	−0.679193	0.3000034	0.229262
Bruxism^b^	18 (26.9%)	22 (24.4%)	12 (18.8%)	0.531	0.216193	−0.13245	−0.025782

*Note:* Data are presented as mean ± SD or *n* (%). Bold indicates statistical significance.

Abbreviations: CMI, craniomandibular index; DI, dysfunction index; PI, palpation index; SD, standard deviation; TMJ, temporomandibular joint; VAS, visual analog scale.

^a^ Continuous variables were analysed using one‐way analysis of variance (ANOVA).

^b^ Categorical variables were analysed using the χ^2^ test. Statistical significance was defined as *p* < 0.05. Regression coefficients were derived from a multiclass logistic regression (one‐vs‐rest) model, in which each effusion grade was compared against the remaining grades and indicate the relative contribution of each variable to each class. Input features were standardized before model fitting; therefore, coefficients should be interpreted as relative model weights rather than causal effect estimates.

* *p* < 0.05.

** *p* < 0.01.

*** *p* < 0.001.

### 3.2 Pain‐Related Characteristics According to TMJ Effusion Presence

Patients with MRI‐detected TMJ effusion showed a significantly greater pain burden than those without effusion. Mean VAS was significantly higher in the effusion group than in the non‐effusion group (5.08 ± 2.16 vs. 3.45 ± 2.09, *p* < 0.001). DI and CMI were also significantly higher in the effusion group (DI: 0.29 ± 0.24 vs. 0.21 ± 0.23, *p* = 0.014; CMI: 0.27 ± 0.26 vs. 0.18 ± 0.24, *p* = 0.025). PI was higher in the effusion group (0.24 ± 0.30 vs. 0.16 ± 0.27, *p* = 0.048), although this difference became borderline after Bonferroni adjustment (adjusted *p* = 0.054). Symptom duration was also significantly longer in the effusion group than in the non‐effusion group (17.25 ± 32.34 vs. 6.34 ± 11.89 months, *p* < 0.001). These between‐group differences are illustrated in Figure 2 and summarized in Table 1.

**FIGURE 2:**
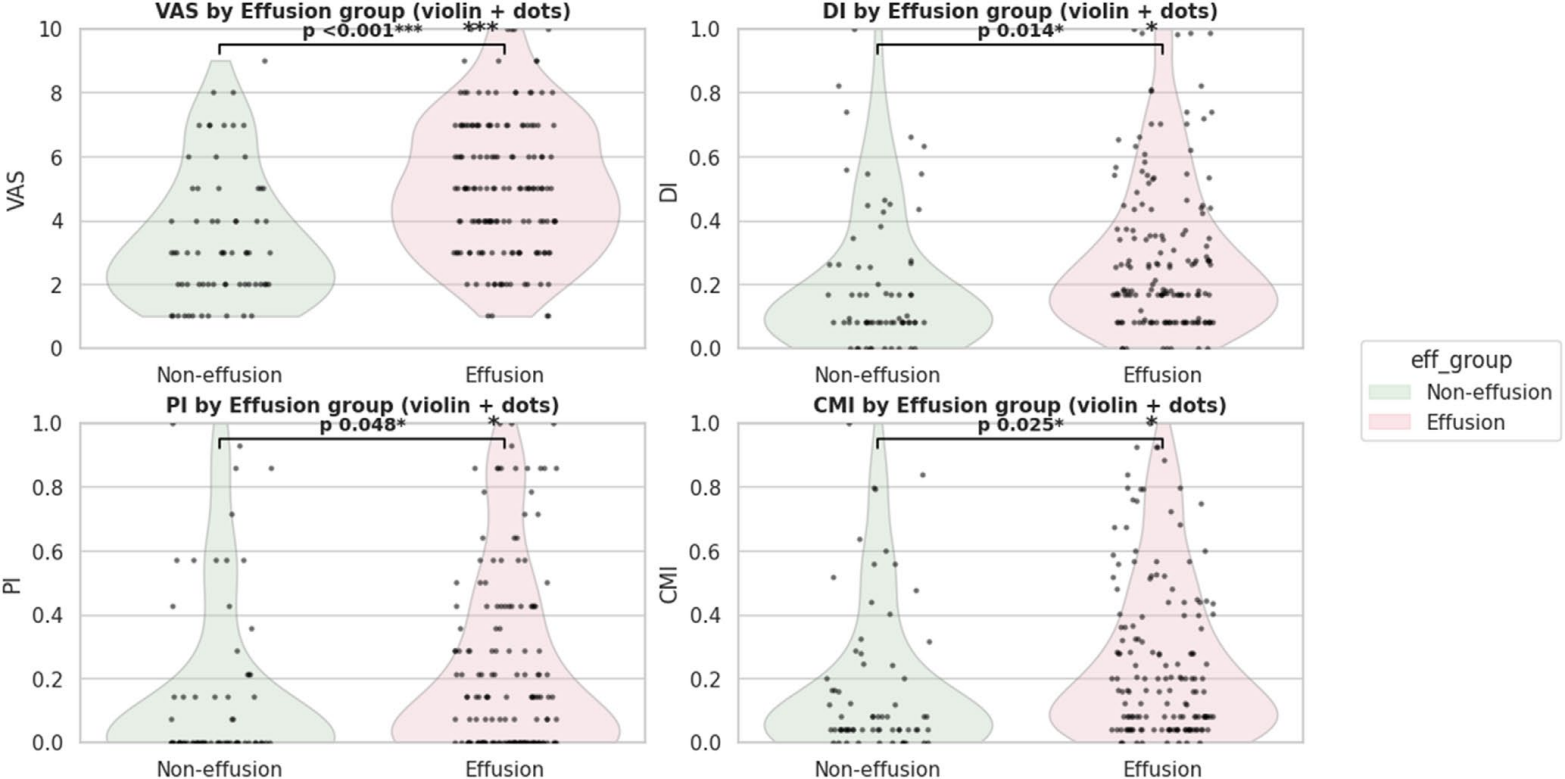
Comparison of pain‐related indices between the non‐effusion and effusion groups. Violin plots overlaid with individual data points illustrate the distribution of VAS, DI, PI, and CMI values according to TMJ effusion status. Each dot represents an individual patient, and the width of each violin reflects the density of the distribution. *p* values for the comparisons between the two groups are indicated above each panel. VAS, visual analog scale; DI, dysfunction index; PI, palpation index; CMI, craniomandibular index.

### 3.3 Clinical Characteristics According to TMJ Effusion Presence

Among associated clinical symptoms, TMJ noise and locking were significantly more frequent in the effusion group than in the non‐effusion group. TMJ noise was present in 68.2% of the effusion group compared with 49.3% of the non‐effusion group (*p* = 0.010), and locking was reported in 50.6% versus 28.4%, respectively (*p* = 0.003). In contrast, muscle stiffness, tinnitus, and bruxism did not differ significantly between the two groups (all *p* > 0.05). These findings indicate that TMJ effusion is associated not only with greater pain intensity and dysfunction but also with selected joint‐related symptoms (Table 1).

### 3.4 Dose–Response Relationship Across TMJ Effusion Severity Grades

When patients were stratified by effusion severity, pain‐related measures showed a clear dose–response pattern. Mean VAS increased progressively from 3.45 ± 2.09 in Grade 0 to 4.72 ± 2.22 in Grade 1 and 5.59 ± 1.99 in Grade 2 (*p* < 0.001). DI also increased across grades (0.21 ± 0.23, 0.25 ± 0.20, and 0.35 ± 0.28, respectively; *p* = 0.003), as did PI (0.16 ± 0.27, 0.19 ± 0.23, and 0.31 ± 0.37; *p* = 0.005) and CMI (0.18 ± 0.24, 0.22 ± 0.21, and 0.33 ± 0.32; *p* = 0.003). Symptom duration also differed significantly across grades (6.34 ± 11.90, 21.71 ± 38.18, and 10.96 ± 20.27 months, respectively; *p* = 0.002), although the pattern was not strictly monotonic. TMJ noise, locking, and tinnitus also varied significantly across grades, whereas age, muscle stiffness, and bruxism did not (Table 2). In the one‐vs‐rest multiclass coefficients, VAS showed the strongest positive contribution to Grade 2 classification, with additional positive contributions from female sex, CMI, TMJ noise, locking, and tinnitus (Table 2).

### 3.5 Correlation Analyses Between TMJ Effusion and Pain‐Related Indices

Correlation analyses further supported the relationship between TMJ effusion and pain burden. As shown in Figure 3, both binary effusion status and ordinal effusion severity were positively associated with VAS, DI, PI, and CMI, with the strongest relationship observed for VAS. The correlation matrix also demonstrated very strong intercorrelations among DI, PI, and CMI, supporting the internal consistency of the dysfunction‐related measures. These findings were consistent with the group‐comparison analyses and further support the interpretation that increasing effusion burden is associated with greater pain and dysfunction (Figure 3).

**FIGURE 3:**
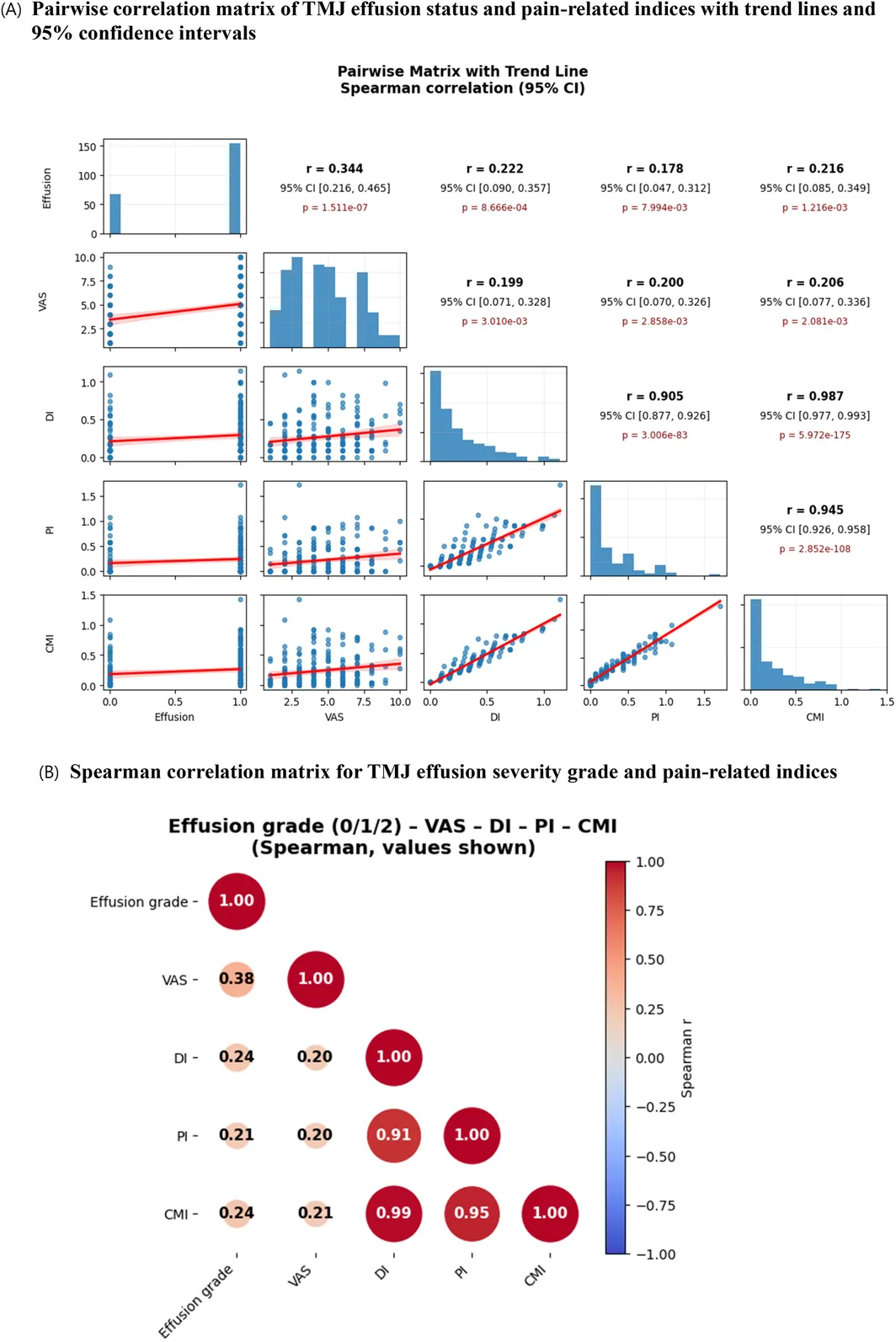
Correlation analyses between TMJ effusion and pain‐related indices: Pairwise correlation matrix of TMJ effusion status and pain‐related indices with trend lines and 95% confidence intervals. (A) Pairwise correlation matrix of TMJ effusion status and pain‐related indices with trend lines and 95% confidence intervals. (B) Spearman correlation matrix for TMJ effusion severity grade and pain‐related indices.

### 3.6 Exploratory Classification Performance for TMJ Effusion Presence and Severity

In the exploratory binary classification analysis for effusion presence, the base model achieved an AUROC of 0.820 (95% CI, 0.651–0.960), and SMOTE modestly increased the AUROC to 0.841 (95% CI, 0.675–0.980), suggesting only limited incremental benefit from class balancing (Figure 4A). In the multiclass one‐vs‐rest analysis, class‐specific AUROCs varied across grades: Grade 0 improved slightly from 0.742 to 0.749, Grade 1 from 0.636 to 0.700, and Grade 2 decreased from 0.599 to 0.570 after SMOTE; none of these differences were statistically significant (Figure 4B). Overall, these exploratory analyses suggest that the model showed moderate discrimination for effusion presence but only limited improvement with oversampling, particularly in the multiclass setting (Figure 4C).

**FIGURE 4:**
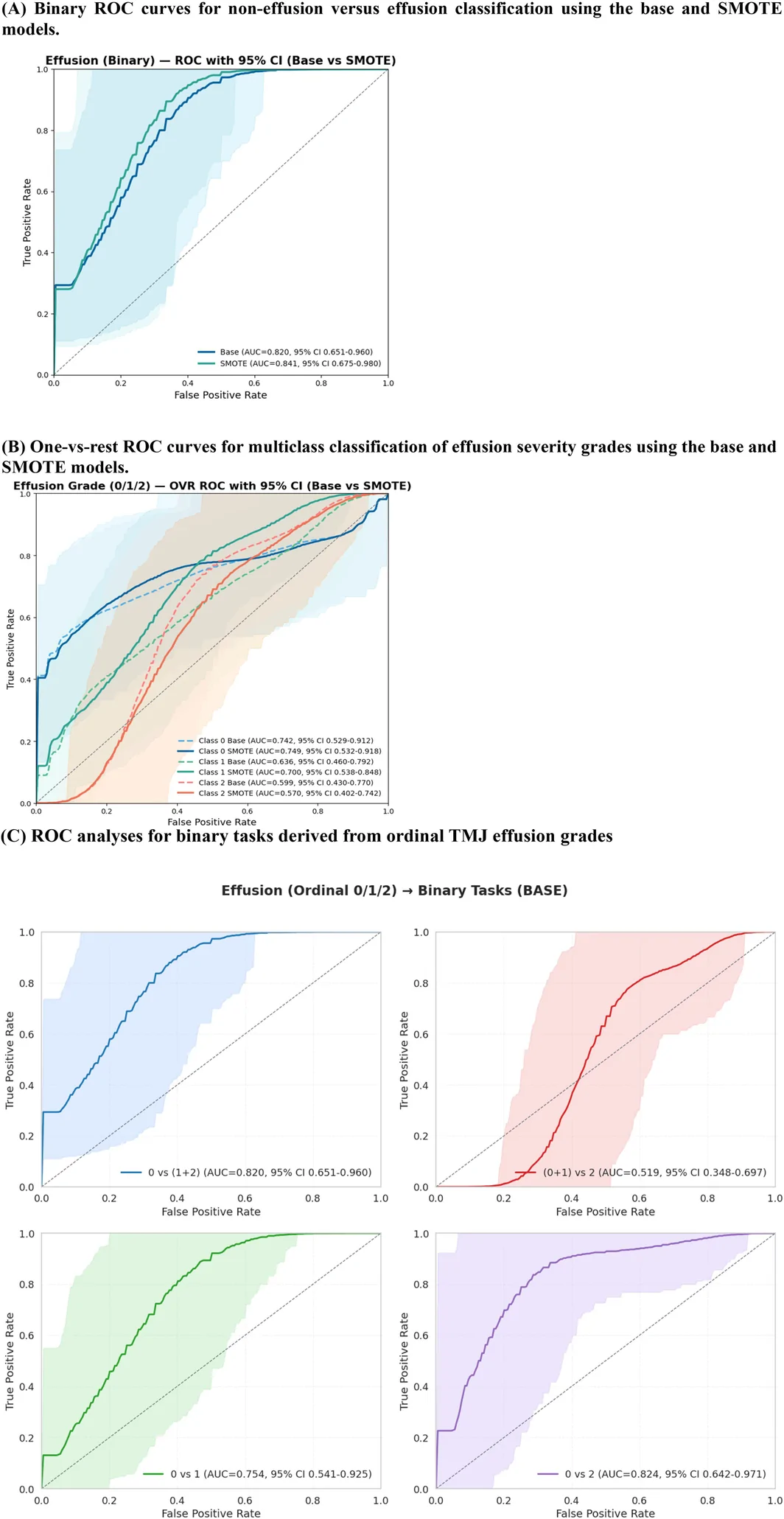
Receiver operating characteristic (ROC) analyses for binary, multiclass, and ordinal‐to‐binary classification of TMJ effusion (A) Binary ROC curves for non‐effusion versus effusion classification using the base and SMOTE models. (B) One‐vs‐rest ROC curves for multiclass classification of effusion severity grades using the base and SMOTE models. (C) ROC analyses for binary tasks derived from ordinal TMJ effusion grades. AUC values and 95% CIs are shown in each panel, and the dashed diagonal line represents chance‐level performance. Abbreviations: ROC, receiver operating characteristic; AUC, area under the curve; CI, confidence interval; SMOTE, synthetic minority over‐sampling technique; OVR, one‐vs‐rest.

### 3.7 Binary‐Task ROC Analyses Derived From Ordinal Effusion Grades

In additional binary tasks derived from ordinal effusion grades, discrimination was strongest for Grade 0 versus Grade 2 (base AUROC 0.824; SMOTE AUROC 0.830) and for Grade 0 versus Grades 1–2 (base AUROC 0.820; SMOTE AUROC 0.841), whereas discrimination for Grades 0–1 versus Grade 2 was poor (base AUROC 0.519; SMOTE AUROC 0.534) (Figure 4C). Across all four binary tasks, SMOTE did not significantly improve AUROC (all DeLong *p* = 1.000; Table S1). Pairwise AUROC comparisons using a common held‐out split also showed no statistically significant differences between selected binary tasks, including Grade 0 versus Grades 1–2 compared with Grades 0–1 versus Grade 2 (ΔAUC = 0.1409, *p* = 0.1484) and Grade 0 versus Grade 2 compared with Grade 0 versus Grade 1 (ΔAUC = −0.0342, *p* = 0.8012) (Figure S1). These results indicate that the model distinguished the extreme groups more effectively than intermediate severity boundaries, but task‐to‐task differences were not statistically significant.

### 3.8 Regression Analyses for TMJ Effusion Presence and Moderate‐To‐Severe Effusion

In univariable logistic regression analyses for TMJ effusion presence, female sex (OR, 2.547; 95% CI, 1.192–5.443; *p* = 0.016), higher VAS (OR, 2.195; 95% CI, 1.478–3.258; *p* < 0.001), higher CMI (OR, 1.545; 95% CI, 1.062–2.249; *p* = 0.023), longer symptom duration (OR, 2.078; 95% CI, 1.044–4.135; *p* = 0.037), TMJ noise (OR, 2.749; 95% CI, 1.324–5.707; *p* = 0.007), locking (OR, 3.017; 95% CI, 1.396–6.523; *p* = 0.005), and tinnitus (OR, 3.663; 95% CI, 1.574–8.520; *p* = 0.003) were significantly associated with effusion presence. In the multivariable model, female sex (OR, 2.778; 95% CI, 1.553–4.969; *p* = 0.001), higher VAS (OR, 2.028; 95% CI, 1.513–2.719; *p* < 0.001), higher CMI (OR, 1.684; 95% CI, 1.259–2.253; *p* < 0.001), TMJ noise (OR, 2.050; 95% CI, 1.171–3.589; *p* = 0.012), locking (OR, 2.448; 95% CI, 1.393–4.301; *p* = 0.002), and tinnitus (OR, 2.280; 95% CI, 1.295–4.016; *p* = 0.004) remained independently associated with TMJ effusion presence, whereas age, symptom duration, muscle stiffness, and bruxism were not independently associated (Table 3; Figure 5A). When moderate‐to‐severe effusion (Grade 2) was compared with Grades 0–1, female sex (OR, 3.287; 95% CI, 1.465–7.377; *p* = 0.004), higher VAS (OR, 2.107; 95% CI, 1.461–3.037; *p* < 0.001), and higher CMI (OR, 1.712; 95% CI, 1.242–2.360; *p* = 0.001) remained significantly associated with Grade 2 effusion, whereas locking showed borderline significance (OR, 2.014; 95% CI, 1.001–4.052; *p* = 0.050) (Table 4; Figure 5). In the coefficient‐based binary classification analysis, the relative weight for discriminating effusion from non‐effusion was highest for VAS (0.817), followed by tinnitus (0.684), symptom duration (0.610), DI (0.428), TMJ noise (0.403), locking (0.380), female sex (0.339), muscle stiffness (0.309), and CMI (0.142) (Table S1). Taken together, these findings indicate that pain severity, dysfunction, and female sex were the most consistent correlates of both effusion presence and greater effusion severity.

**TABLE 3:** Univariable and multivariable logistic regression analyses of factors associated with the presence of tMJ effusion.

Parameters	Analysis 1: Univariable logistic regression analysis for effusion presence (Grades 1–2 vs. Grade 0)				Analysis 2: Multivariable logistic regression analysis with backward selection for effusion presence (Grades 1–2 vs. Grade 0)			
	OR	Lower	Upper	*p*	OR	Lower	Upper	*p*
		95% CI	95% CI			95% CI	95% CI	
**Age**	0.923	0.652	1.306	0.650	0.935	0.718	1.218	0.619
**Sex (Female)**	2.547	1.192	5.443	**0.016** *	2.778	1.553	4.969	**0.001** **
**VAS**	2.195	1.478	3.258	**< 0.001** ***	2.028	1.513	2.719	**< 0.001** ***
**CMI**	1.545	1.062	2.249	**0.023** *	1.684	1.259	2.253	**< 0.001** ***
**Symptom duration**	2.078	1.044	4.135	**0.037** *	1.046	0.815	1.341	0.725
**TMJ noise**	2.749	1.324	5.707	**0.007** **	2.05	1.171	3.589	**0.012** *
**Locking**	3.017	1.396	6.523	**0.005** **	2.448	1.393	4.301	**0.002** *
**Muscle stiffness**	1.788	0.862	3.712	0.119	1.531	0.883	2.653	0.129
**Tinnitus**	3.663	1.574	8.52	**0.003** **	2.28	1.295	4.016	**0.004** **
**Bruxism**	0.635	0.273	1.48	0.293	0.727	0.383	1.381	0.330

*Note:* Odds ratios (ORs), 95% confidence intervals (CIs), and *p* values are presented. The dependent variable was TMJ effusion presence, defined as effusion (Grades 1–2) versus non‐effusion (Grade 0). Analysis 1 was performed using univariable binary logistic regression to assess the association between each variable and TMJ effusion presence. Analysis 2 was performed using multivariable binary logistic regression with backward selection to identify factors independently associated with TMJ effusion presence. An OR > 1 indicates a higher likelihood of belonging to the effusion group, whereas an OR < 1 indicates a lower likelihood. Bold indicates statistical significance (*p* < 0.05).

Abbreviations: CI, confidence interval; CMI, craniomandibular index; OR, odds ratio; TMJ, temporomandibular joint; VAS, visual analog scale.

* *p* < 0.05.

** *p* < 0.01.

*** *p* < 0.001.

**FIGURE 5:**
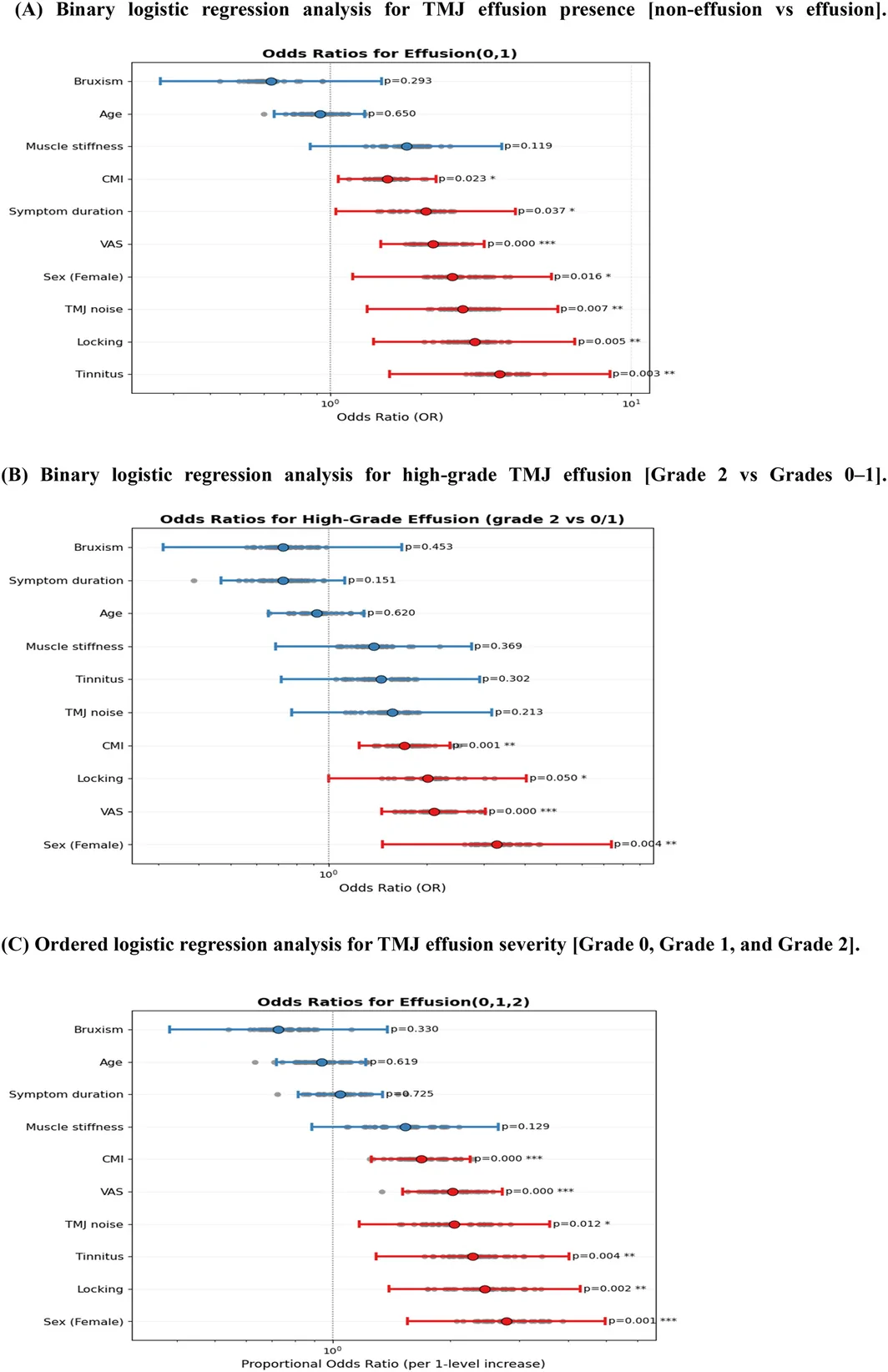
Forest plots of regression analyses for TMJ effusion presence and severity. (A) Binary logistic regression analysis for TMJ effusion presence [non‐effusion vs effusion]. (B) Binary logistic regression analysis for high‐grade TMJ effusion [Grade 2 vs Grades 0–1]. (C) Ordered logistic regression analysis for TMJ effusion severity [Grade 0, Grade 1, and Grade 2]. The vertical dashed line indicates OR = 1.0, representing no association. Variables shown in red indicate statistically significant associations (*p* < 0.05), whereas variables shown in blue indicate non‐significant associations. Abbreviations: OR, odds ratio; CI, confidence interval; VAS, visual analog scale; CMI, craniomandibular index; TMJ, temporomandibular joint.

**TABLE 4:** Univariable and multivariable logistic regression analyses of factors associated with moderate‐to‐severe tMJ effusion (Grade 2) versus Grades 0–1.

Parameters	Univariable logistic regression analysis for moderate‐to‐severe effusion (Grade 2 vs Grade 0–1)				Analysis 2: Multivariable regression analysis with backward selection moderate‐to‐severe effusion (Grade 2 vs. Grade 0–1)			
	OR	Lower	Upper	*p*	OR	Lower	Upper	*p*
		95% CI	95% CI			95% CI	95% CI	
**Age**	1.028	0.769	1.373	0.854	0.918	0.665	1.287	0.620
**Sex (Female)**	2.427	1.219	4.832	**0.012** **	3.287	1.465	7.377	**0.004** **
**VAS**	1.906	1.396	2.601	**< 0.001** ***	2.107	1.461	3.037	**< 0.001** ***
**CMI**	1.574	1.184	2.094	**0.002** **	1.712	1.242	2.36	**0.001** **
**Symptom duration**	0.836	0.587	1.192	0.323	0.726	0.469	1.124	0.151
**TMJ noise**	1.474	0.795	2.734	0.218	1.568	0.773	3.178	0.213
**Locking**	1.691	0.942	3.037	**0.079** *	2.014	1.001	4.052	**0.049** *
**Muscle stiffness**	1.640	0.913	2.946	0.098	1.374	0.687	2.75	0.369
**Tinnitus**	1.404	0.763	2.586	0.276	1.446	0.718	2.914	0.302
**Bruxism**	0.675	0.328	1.391	0.287	0.724	0.311	1.684	0.453

*Note:* Odds ratios (ORs), 95% confidence intervals (CIs), and *p* values are presented. The dependent variable was moderate‐to‐severe TMJ effusion (Grade 2), with Grades 0–1 used as the reference group. Analysis 1 was performed using univariable binary logistic regression to assess the association between each variable and Grade 2 TMJ effusion. Analysis 2 was performed using multivariable binary logistic regression with backward selection to identify factors independently associated with Grade 2 TMJ effusion. An OR > 1 indicates a higher likelihood of belonging to the Grade 2 effusion group, whereas an OR < 1 indicates a lower likelihood. Bold indicates statistical significance (p < 0.05).

Abbreviations: CI, confidence interval; CMI, craniomandibular index; OR, odds ratio; TMJ, temporomandibular joint; VAS, visual analog scale.

* *p* < 0.05.

** *p* < 0.01.

*** *p* < 0.001.

### 3.9 Sensitivity Analysis: Concomitant MRI Findings

ADD and OA were present in 162 (73.3%) and 157 (71.0%) patients, respectively. Both findings were associated with effusion status (ADD, *p* = 0.012, Cramér's V = 0.169; OA, *p* = 0.009, Cramér's V = 0.176), although the associations were modest. Neither ADD nor OA was independently associated with VAS or CMI (all *p* > 0.29). After ADD and OA were added to the regression models, the association between effusion and pain/dysfunction remained essentially unchanged: the effusion coefficient for VAS changed from 1.69 (95% CI, 1.07–2.31) to 1.62 (95% CI, 0.98–2.26), and the coefficient for CMI changed from 0.090 (95% CI, 0.015–0.164) to 0.095 (95% CI, 0.018–0.172), with both remaining statistically significant. Comparable results were obtained when effusion was modelled as grade. All VIFs were below 1.3 (Table S2).

### 3.10 Pain and Dysfunction by Effusion Laterality

Among patients with effusion, 60 had unilateral and 94 had bilateral involvement. Bilateral effusion was not associated with higher pain or dysfunction than unilateral effusion (VAS, 5.15 vs. 4.98; DI, 0.28 vs. 0.31; PI, 0.23 vs. 0.25; CMI, 0.26 vs. 0.28; all *p* > 0.68; Table S3 and Figure S2). In the three‐group comparison, VAS, DI, PI, and CMI differed significantly among patients with no effusion, unilateral effusion, and bilateral effusion (Kruskal–Wallis test: VAS, *p* < 0.001; DI, *p* = 0.004; PI, *p* = 0.031; CMI, *p* = 0.006). These differences appeared to reflect the presence of effusion rather than a progressive increase from unilateral to bilateral involvement.

## 4 Discussion

In this study, severity‐graded MRI‐detected TMJ effusion was associated with greater pain intensity and mandibular dysfunction in patients with TMJ arthralgia. This association was evident not only when effusion was analysed as absent versus present, but also when effusion was examined across ordinal severity grades. Although previous studies have demonstrated an association between TMJ effusion and pain [19, 25], relatively few have simultaneously evaluated the degree of functional limitation and broader clinical characteristics. These findings support the interpretation that TMJ effusion represents a clinically meaningful continuum of intra‐articular abnormality rather than a simple all‐or‐none radiologic finding.

A notable finding of this study is that several factors were consistently associated with both TMJ effusion presence and greater effusion severity. Female sex, higher pain intensity, and greater dysfunction emerged as the most robust correlates across the regression models. Although greater pain severity in women than in men has been well documented in patients with TMD [26, 27], evidence directly linking this sex‐related difference to MRI‐detected structural abnormalities of the TMJ remains limited. By contrast, TMJ noise, locking, and tinnitus were independently associated with effusion presence but were less consistently retained in the severity‐focused model. Effusion presence may represent a broader symptomatic and mechanical joint phenotype [28], whereas more severe effusion may reflect a more advanced intra‐articular state more closely linked to pain intensity and functional limitation [8, 29]. The persistence of VAS and CMI as significant variables across both presence‐ and severity‐based analyses strengthens the view that TMJ effusion is not simply an incidental imaging finding, but rather a clinically relevant marker of pain and dysfunction.

The relationship between TMJ effusion and TMD pain is biologically plausible. Mechanistically, TMJ effusion is generally understood to reflect an imbalance between synovial fluid production and clearance and may arise in the setting of intra‐articular inflammation, increased vascular permeability, and biochemical alterations within the joint environment [6]. Such a milieu may contribute to nociceptor sensitization and pain amplification through inflammatory mediators, increased intra‐articular pressure, and capsular distension [30]. In this context, the present findings support the view that TMJ effusion may represent an imaging correlate of an active intra‐articular process rather than a purely incidental fluid signal [31]. At the same time, our results also suggest that effusion alone is unlikely to fully account for clinical pain. The non‐monotonic pattern observed for symptom duration, together with the finding that some variables were associated with effusion presence but not with Grade 2 status, indicates that TMJ pain likely arises from a more complex interaction among inflammatory, mechanical, structural, and patient‐level factors.

Our findings may help explain why previous studies on TMJ effusion and pain have yielded inconsistent conclusions. This variability likely reflects differences in diagnostic frameworks, MRI protocols, effusion definitions and grading criteria, imaging timing relative to symptom fluctuations, and coexisting intra‐articular abnormalities such as disc displacement and osteoarthritic change. Classic MRI‐based studies provide important historical context: Güler et al. linked joint pain with MRI‐graded effusion and total protein concentration in synovial fluid, suggesting that imaging‐detected effusion may reflect both symptomatic and biochemical aspects of the intra‐articular environment [32]. Costa et al. also reported associations among TMJ internal derangement, headache, bruxism, joint pain, and MRI‐detected joint effusion [33]. Together, these studies support interpreting TMJ effusion within the broader spectrum of internal derangement and symptomatic joint pathology rather than as an isolated radiologic sign. Nevertheless, earlier studies also suggested that pain alone is not a reliable predictor of effusion and that effusion may not independently determine pain across all clinical subgroups [14, 34, 35]. Similarly, Khawaja et al. found no significant association between joint effusion and DC/TMD arthralgia or pain‐related clinical characteristics, despite observing relationships between effusion and disc position [6]. By contrast, recent syntheses have been more supportive of a pain–effusion link, reporting that MRI‐detected effusion is frequently associated with pain in symptomatic TMJ studies [36, 37]. The present study extends this evidence by showing that severity‐graded effusion is associated with pain and dysfunction even after considering concomitant ADD and OA.

In addition, non‐contrast MRI cannot always reliably distinguish simple joint fluid from synovial thickening, which complicates mechanistic interpretation. Although MRI is clinically valuable for TMJ assessment, joint effusion should be interpreted in conjunction with other structural findings rather than in isolation [38]. Effusion visualized on T2‐weighted MRI has been suggested to correlate with synovial inflammatory activity, supporting the view that effusion may reflect synovitis rather than a purely incidental fluid signal [39]. Previous studies have also highlighted the close relationship between effusion and internal derangement: Roh et al. reported substantially increased odds of effusion in joints with anterior disc displacement, especially without reduction [40], and Yasan et al. found that higher‐grade effusion was associated with anterior disc displacement, disc deformity, and osteophyte formation [41].

These observations suggest that TMJ effusion is embedded within a broader intra‐articular pathologic spectrum rather than representing an isolated imaging abnormality. Consistent with this view, adjustment for concomitant ADD and OA did not materially alter the effusion–pain/dysfunction association in the present study, and the laterality analysis supported patient‐level grouping of unilateral and bilateral effusion as effusion‐present cases (Table S2 and Table S3). Overall, the dose–response relationship observed across effusion grades supports the interpretation that more advanced effusion may better reflect the burden of active intra‐articular dysfunction and pain‐related joint pathology.

This study has several methodological strengths. First, it included a relatively well‐characterized cohort of TMJ arthralgia patients diagnosed using DC/TMD‐based criteria and evaluated with standardized MRI protocols. Second, effusion was assessed using both T2‐weighted and proton density–weighted sequences, and a structured severity grading system was applied at the patient level. Third, the study examined pain intensity, dysfunction‐related indices, symptom duration, and associated clinical features, allowing a broader assessment of the clinical phenotype linked to effusion. Finally, the integration of conventional statistical comparisons, regression analyses, and exploratory classification analyses provided complementary information on clinical associations, severity patterning, and relative feature contribution. However, the exploratory classification analyses should be interpreted cautiously. Although the models showed moderate discrimination for effusion presence and better separation between extreme severity categories, these findings were derived from a single‐center retrospective cohort using an internal train–test split and a limited set of routinely collected clinical variables. Therefore, the results should not be interpreted as evidence of a clinically deployable screening or diagnostic tool. Rather, their value is descriptive and supportive: they identify clinical features that contributed most to effusion discrimination and reinforce the severity gradient observed in the conventional clinical analyses. External validation in larger independent cohorts, with calibration assessment and prospective testing, would be required before any translational application can be considered.

Several limitations should be acknowledged. First, the cross‐sectional and retrospective design precludes causal inference regarding whether effusion contributes directly to pain generation, whether pain‐related joint loading promotes effusion, or whether both arise from a shared intra‐articular process. Second, this was a single‐center study, which may limit generalizability to other populations and clinical settings. Third, although MRI is well suited for evaluating intra‐articular fluid, non‐contrast MRI cannot fully separate fluid accumulation from synovial thickening or active synovitis. Fourth, patient‐level severity was determined using the higher grade in cases of bilateral effusion, which is clinically practical but may simplify joint‐level heterogeneity. Fifth, although coexisting anterior disc displacement and osteoarthritic change were evaluated and did not materially alter the effusion–pain/dysfunction association, bone marrow edema was not available for analysis, and broader psychosocial contributors were not comprehensivelymodelled. Finally, the exploratory classification analyses require external validation before any clinical application can be considered.

## 5 Conclusion

Severity‐graded MRI‐detected TMJ effusion was associated with greater pain intensity and mandibular dysfunction in patients with TMJ arthralgia. This association was observed across both binary and ordinal effusion analyses and was not materially explained by concomitant ADD or OA. These findings support the interpretation of TMJ effusion severity as a clinically meaningful imaging marker rather than an incidental fluid signal. Future longitudinal studies with repeated symptom assessment and serial imaging are needed to determine whether changes in effusion parallel changes in pain over time and whether effusion predicts symptom persistence, fluctuation, or worsening. Further work should also integrate additional structural and biological markers, including disc position, osteoarthritic change, bone marrow edema, and more direct indicators of synovial inflammation.

## Author Contributions

**Yung‐Kyun Noh:** supervision, software, methodology, conceptualization, funding acquisition, writing – original draft, writing – review and editing, project administration. **Seonggwang Jeon:** writing – original draft, writing – review and editing, software, methodology, validation, investigation, visualization, formal analysis, data curation. **Seong‐Woo Jang:** software, formal analysis, methodology, validation, visualization, writing – review and editing, writing – original draft, data curation, resources. **Q‐Schick Auh:** writing – original draft, writing – review and editing, data curation, resources, validation, investigation. **Yeon‐Hee Lee:** conceptualization, investigation, writing – original draft, methodology, validation, visualization, writing – review and editing, software, formal analysis, project administration, data curation, resources, supervision.

## Funding

This work was supported by a National Research Foundation of Korea (NRF) grant funded by the Ministry of Science and ICT (MSIT) (RS‐2024‐00421203), and by grants from the Institute of Information & Communications Technology Planning & Evaluation (IITP) funded by the MSIT (IITP‐2021‐0‐02068; RS‐2020‐II201373; RS‐2023‐00220628).

## Conflicts of Interest

The authors declare no conflicts of interest.

## Supporting information


**Table S1:** Regression coefficients for variables associated with TMJ effusion compared with non‐effusion.
**Table S2:** Association between joint effusion and pain severity before (Model 1) and after (Model 2) adjustment for concomitant MRI findings (anterior disc displacement and osteoarthritic change).
**Table S3:** Pain and dysfunction indices by effusion laterality.
**Figure S1:** Pairwise AUROC comparisons between selected binary tasks using a common held‐out split.
**Figure S2:** Pain and dysfunction indices by effusion laterality.

## Data Availability

The data that support the findings of this study are openly available in GitHub_TMJ‐Effusion‐and‐Pain at https://github.com/seongwoojang1123/TMJ‐Effusion‐and‐Pain.git.
