# Supplementary material for: Severity‐Graded MRI‐Detected TMJ Effusion Is Associated With Pain and Dysfunction in Temporomandibular Disorder Patients
Source: J Oral Rehabil. 2026 Jul 25;53(12):2266–81. doi: 10.1111/joor.70276 (PMC13755669; doi:10.1111/joor.70276)
Supplement: Supplementary file 1 [file JOOR-53-2266-s001.docx]

**Supplementary Table 1. Regression coefficients for variables associated with TMJ effusion compared with non-effusion**

| **Variable** | **Coefficient** |
| --- | --- |
| VAS | 0.817047 |
| Tinnitus | 0.684447 |
| Symptom duration | 0.610308 |
| DI | 0.427746 |
| TMJ noise | 0.402984 |
| Locking | 0.379606 |
| Sex (Female) | 0.33926 |
| Muscle stiffness | 0.308518 |
| CMI | 0.141804 |
| PI | -0.093132 |
| Age | -0.154678 |
| Bruxism | -0.222646 |

Regression coefficients were obtained from a binary logistic regression model in which the non-effusion group was used as the reference category. Positive coefficient values indicate that the variable is more strongly associated with the effusion group, whereas negative values indicate a stronger association with the non-effusion group. Variables are presented in descending order of coefficient magnitude. Input features were standardized before model fitting; therefore, coefficients should be interpreted as relative model weights rather than causal effect estimates. VAS, visual analog scale; DI, dysfunction index; PI, palpation index; CMI, craniomandibular index; TMJ, temporomandibular joint.

**Supplementary Figure 1. Pairwise AUROC comparisons between selected binary tasks using a common held-out split**

| **Task_A** | **Task_B** | **AUC_A** | **AUC_B** | **95%_CI_L** | **95%_CI_U** | **Δ AUC** | **p-value** |
| --- | --- | --- | --- | --- | --- | --- | --- |
| **Grade 0 vs (Grade 1+2)** | Grade (0+1) vs Grade 2 | 0.744 | 0.603 | -0.0502 | 0.3318 | 0.1409 | 0.1484 |
| **Grade 0 vs Grade 2** | Grade 0 vs Grade 1 | 0.719 | 0.754 | -0.3177 | 0.2618 | -0.0342 | 0.8012 |

**Supplementary Table 2. Association between joint effusion and pain severity before (Model 1) and after (Model 2) adjustment for concomitant MRI findings (anterior disc displacement and osteoarthritic change).**

| **Pain outcome** | **Predictor** | **Model 1 (base): β (95% CI)** | **p-value** | **Model 2 (+ ADD + OA): β (95% CI)** | **p-value** |
| --- | --- | --- | --- | --- | --- |
| **Effusion modeled as presence (absent vs. present)** | | | | | |
| VAS | Effusion | 1.69 (1.07, 2.31) | <0.001 | 1.62 (0.98, 2.26) | <0.001 |
|  | ADD | — | — | 0.07 (−0.61, 0.74) | 0.848 |
|  | OA | — | — | 0.35 (−0.31, 1.01) | 0.297 |
| CMI | Effusion | 0.090 (0.015, 0.164) | 0.018 | 0.095 (0.018, 0.172) | 0.015 |
|  | ADD | — | — | −0.033 (−0.114, 0.048) | 0.424 |
|  | OA | — | — | 0.002 (−0.077, 0.081) | 0.967 |
| **Effusion modeled as grade (per 1-grade increment)** | | | | | |
| VAS | Effusion grade | 1.13 (0.76, 1.50) | <0.001 | 1.09 (0.72, 1.47) | <0.001 |
| CMI | Effusion grade | 0.080 (0.036, 0.124) | <0.001 | 0.082 (0.037, 0.128) | <0.001 |

Values are unstandardized regression coefficients (β) with 95% confidence intervals from linear regression (n = 221). Model 1 is adjusted for age and sex; Model 2 additionally includes ADD and OA (both coded present/absent). VAS, visual analog scale; CMI, craniomandibular index. Bone marrow edema was not available and was not modeled. All variance inflation factors were < 1.3.

**Supplementary Table 3. Pain and dysfunction indices by effusion laterality.**

| **Index** | **No effusion (n=67)** | **Unilateral (n=60)** | **Bilateral (n=94)** | **p-value (uni vs. bi)** | **p-value (3-group)** |
| --- | --- | --- | --- | --- | --- |
| VAS | 3.45 ± 2.09 | 4.98 ± 2.19 | 5.15 ± 2.16 | 0.683 | <0.001 |
| DI | 0.21 ± 0.23 | 0.31 ± 0.28 | 0.28 ± 0.21 | 0.876 | 0.004 |
| PI | 0.16 ± 0.27 | 0.25 ± 0.35 | 0.23 ± 0.28 | 0.954 | 0.031 |
| CMI | 0.18 ± 0.24 | 0.28 ± 0.30 | 0.26 ± 0.23 | 0.763 | 0.006 |

Values are mean ± SD. P-value (uni vs. bi): Mann–Whitney U test, unilateral versus bilateral. P-value (3-group): Kruskal–Wallis test across no effusion, unilateral, and bilateral. VAS, visual analog scale; DI, dysfunction index; PI, palpation index; CMI, craniomandibular index.

**Supplementary Figure 2. Pain and dysfunction indices by effusion laterality**

Pain (VAS) and dysfunction indices (DI, PI, CMI) by effusion laterality. Boxes show median and interquartile range; points are individual patients. Brackets indicate the unilateral-versus-bilateral comparison (Mann–Whitney U); the three-group Kruskal–Wallis result is annotated in each panel. VAS, visual analog scale; DI, dysfunction index; PI, palpation index; CMI, craniomandibular index.
